# Child protection outcomes of the Australian Nurse Family Partnership Program for Aboriginal infants and their mothers in Central Australia

**DOI:** 10.1371/journal.pone.0208764

**Published:** 2018-12-07

**Authors:** Leonie Segal, Ha Nguyen, Debra Gent, Catherine Hampton, John Boffa

**Affiliations:** 1 Health Economics and Social Policy Group, School of Health Sciences, University of South Australia, Adelaide, South Australia, Australia; 2 Central Australian Aboriginal Congress Aboriginal Corporation, Alice Springs, Northern Territory, Australia; Harvard TH Chan School of Public Health, UNITED STATES

## Abstract

**Background:**

The Nurse Family Partnership Program developed in the USA, designed to improve mother and infant/child outcomes, has reported lower rates of child protection system involvement. The study tested the hypothesis that an adapted Nurse Family Partnership Program implemented in an Aboriginal community in Central Australia (the FPP) would improve Child Protection outcomes.

**Methods:**

This was a retrospective and prospective cohort study drawing on linked administrative data, including birth registry, primary health care client information system, FPP program data, and child protection data. Participants were children of women eligible for the FPP program (an exposed and a control group of women, eligible but not referred) live-born between 1/3/2009 (program commencement) and 31/12/2015. Child protection data covered all reports, investigations, substantiations and out-of-home care placements from the time of the child’s birth to 31/12/2016. Generalised linear modelling was used to estimate the relative risk (RR) of involvement with child protection and type of involvement (report, investigation, substantiation, out-of-home-care placement) among FPP and control children.

**Results:**

FPP mothers (n = 291) were on average younger, were more likely to be first-time mothers and experiencing housing instability than control mothers (n = 563). Among younger mothers ≤20 years, FPP children had statistically significantly lower rates of involvement with child protection (ARR_report_ = 0.49, 95% CI: 0.29 to 0.82; ARR_investigation_ = 0.34, 95% CI: 0.19 to 0.64; ARR_substantiation_ = 0.45, 95% CI: 0.21 to 0.96) and experience fewer days in care (ARR = 0.10, 95% CI: 0.02 to 0.48). Among children of first-time mothers, FPP children also had statistically significantly lower rates of involvement with child protection (ARR_report_ = 0.50, 95% CI: 0.30 to 0.83; ARR_investigation_ = 0.36, 95% CI: 0.19 to 0.67; ARR_substantiation_ = 0.38, 95% CI: 0.18 to 0.80) and fewer days in care (ARR = 0.06, 95% CI: 0.01 to 0.27).

**Conclusion:**

Study results suggest a modified Nurse Family Partnership delivered by an Indigenous community-controlled organisation may have reduced child protection system involvement in a highly vulnerable First Nations population, especially in younger or first-time mothers. Testing these results with an RCT design is desirable.

## Introduction

Child abuse and neglect among Aboriginal and Torres Strait Islander populations (hereafter referred to as Aboriginal) is a particular concern in Australia, with Aboriginal children significantly over-represented in the child protection system. In 2015–16, the rates of substantiation and child removal to out-of-home care among Aboriginal children were respectively, nearly seven times (43.6/1,000 vs. 6.4/1,000) and nearly ten times (56.6/1,000 vs. 5.8/1,000) those of non-Aboriginal children [1].

The extreme disadvantage experienced by much of the Aboriginal population, such as deep poverty, racism, insecure housing, poor nutrition, parental separation, poor mental health, drug and alcohol problems, low educational attainment, high unemployment, welfare dependency and chronic illness is implicated in high rates of child abuse and neglect [2,3]. These vulnerabilities have their genesis in the complex trauma histories of Aboriginal people, of colonisation and dispossession, racism and child removal, compounded by family and community violence, incarceration and premature death. The complex nature of risk exposure in Aboriginal communities and the seriousness of consequences of multiple adversities underline the need for effective family support services to mitigate the negative consequences and potentially disrupt intergenerational pathways into profound disadvantage. The National Framework for Protecting Australia’s Children 2009–2020 stresses the need of early interventions to address family issues early in a child’s life [4].

### Home visiting programs

Home visiting programs have emerged as a promising prevention strategy for vulnerable families, delivering support to pregnant women and their infants in their homes. A home-based service is designed to increase access, provide opportunities to involve family members in an environment that supports the building of a trusting relationship between mothers and nurse-visitors [5,6]. The goal is that by intervening early in the infant’s life, typically during pregnancy, new parents will be assisted in establishing a nurturing parenting style to achieve specific program objectives. Programs differ in terms of the type of families served (such as teenage mothers, first-time mothers, particular ethnicities); targeted outcomes (such as child maltreatment, child development, mother’s employment); the underpinning theory of change; qualification of home visitor; the types of services and support offered and the length and intensity of home visit schedule [5–7].

### Nurse home visiting program in Central Australia

The Australian government, in an initiative designed for Aboriginal families to improve pregnancy outcomes, enhance child health and well-being and assist mothers in their own development, funded the delivery of an adapted model of the Olds Nurse Family Partnership (NFP) for mothers of Aboriginal children (dubbed the ANFPP) [8]. The NFP was chosen as one of a small number of home-visiting programs with evidence of capacity to reduce child abuse and neglect and improve other outcomes [9].

The initiative was promoted as a community-level early investment in the future of Aboriginal children. Program delivery commenced at three sites in 2009, in Alice Springs (and surrounds), Central Australia (Northern Territory), at Wellington (New South Wales) and Cairns (Queensland). By mid-2017, the ANFPP had been expanded to an additional three sites across the country, and to a further seven sites by June 30 2018 [10]. Across all ANFPP sites Aboriginal community workers were included as part of the home visiting team, the primary modification on the NFP model.

To date there have been no published outcome evaluations of any of the ANFPP sites, or more broadly of infant home visiting for a remote first nations population. The aim of this study was to examine the effect of the Central Australian Family Partnership Program (FPP) on child protection outcomes among children in the program, specifically on the rates of child protection reports, investigations, substantiations and the annualised number of days in out-of-home care.

The FPP was delivered by a large Aboriginal community-controlled health service in Central Australia (hereafter referred to as Central Australia health service). A decision was made to deliver the program to multiparas women, not just first-time mothers, a departure from the NFP. The service did not wish to limit access to the program, which was hoped would benefit Aboriginal mothers and their infants.

Referral pathways into the FPP were not restricted. All pregnant women who met the inclusion criteria of i) location in the town of Alice Springs between 10 and 22 weeks gestation, ii) the expectant mother (or father) was Aboriginal, iii) the mother had not previously participated in the FPP were eligible. Most referrals came through the Central Australian Health Service midwifery program. But self-referral and referrals from other services also occurred [11]. Reasons for non-referral are not known (but may include turnover of staff and lack of knowledge of the FPP, or a view that given access to maternity services for all pregnant women, it was not important).

## Method

### Study design

This evaluation adopted a retrospective and prospective comparative cohort design, adjusting for pertinent base-line differences. The outcome of interest was involvement with the child protection system (CPS) as a measure of child maltreatment. Study design was constrained by the context—program evaluation proceeding alongside service delivery in a remote community setting, commencing after service delivery had begun. Furthermore, Mr Nick Pascual, Director of Child and Family Health, Australian Department of Health (Segal L 2017, telephone conversation, 13^th^ June) advised that our plan to conduct a randomised control trial (RCT) of the ANFPP could not proceed due to rejection of the RCT design by an Aboriginal Health Research Ethics Committee.

Our study was approved by the Research Steering Committee of the Central Australian Aboriginal Congress Aboriginal Corporation, the Human Research Ethics Committee of the University of South Australia (protocol 32377) and the Central Australian Human Research Ethics Committee (HREC-14-205).

### Participants

Participants included all live-born children to women on the Health service data-base born between 01/03/2009 (program commencement) and 31/12/2015 who met the eligibility criteria for the FPP of: i) pregnant with an Aboriginal baby; ii) recorded locality during pregnancy (13 to 28 weeks gestation) was within the program catchment (Alice Springs and within 100 km radius); iii) not having an early miscarriage, termination or abortion (<13 weeks gestation); and iv) had not previously enrolled in the program [8]. Children’s live-born status was sourced from the Health service’s Patient Information and Records System (PIRS). Two study cohorts were created: i) an exposed cohort (the FPP group) of all children live-born to women who had enrolled in the FPP and ii) the control group, children of eligible women who were not referred to and had never participated in the FPP. Eligible women who were referred but declined to participate were excluded from the analysis altogether; they did not form part of our control or FPP group.

### Data sources and data items

De-identified datasets used for the study were derived from the PIRS, the Northern Territory Birth Registry and the Northern Territory Child Protection. An independent data linkage unit (SANT DataLink, an operational unit established to link administrative data in South Australia and the Northern Territory) was engaged to de-identify all datasets, assigning a unique project specific linkage key to all children across the three data sources. The research team only had access to de-identified data.

PIRS was the data source for Health service clients’ Aboriginal status, localities during pregnancy, pregnancy outcome and FPP enrolment status, which were used to determine eligibility for the FPP. Clients’ localities were also used to describe their level of vulnerability, measured by the number of locality changes (or house moves within two years of being eligible/enrolled into the FPP) and area socio-economic quintile at the time of the child’s birth. The coding of localities, as “in” or “out” of the program catchment area, was completed by staff from the Central Australia Health service, and client’s locality at the time of the child’s birth was extracted and provided to an independent geo-spatial coder to allocate a socioeconomic index using the Index of Relative Socio-economic Advantage and Disadvantage (IRSAD) [12]. These were then grouped into quintiles, with Q1 the most disadvantaged and Q5 the least disadvantaged (most advantaged).

The Northern Territory Birth Registry provided descriptive variables that represented potential confounding factors or moderators of program outcome–specifically the number of previous live-born children of each mother and mother’s employment status at the time of the child’s birth, for all FPP and control children.

The Northern Territory Child Protection (CP) data contains complete records of involvement with the statutory child protection system in the Northern Territory. Records covered all child protection reports, investigations, substantiations and out-of-home care placements from the time of the child’s birth to 31/12/2016.

### Measurement/Definition

A child protection report is recorded in the NT child protection administrative system when a member of the community (including health, education, community professionals, police, neighbours, relatives) makes a report or notification to the statutory child protection department to signify that they have reason to believe that a child is at risk of harm and in need of protection [13]. The focus of reports to CP is familial abuse and neglect (not extra-familial which, as a criminal offence, is a police matter).

A child protection investigation is commenced if a CP report reaches a threshold of concern. During a CP investigation the department obtains more information about the child who is the subject of a report and makes an assessment about the risk of child abuse or neglect, likelihood of harm and the child’s protective needs. Information may be gathered by checking information systems, undertaking discussions with agencies and individuals, interviewing/sighting the child and/or interviewing the caregivers/parents [13].

A child protection substantiation, as an outcome of an investigation, means that there is reason to believe a child has been, is being or is likely to be abused, neglected and subject to serious harm and an appropriate intervention by child protection services will be determined. An active response can include referral to family support or other services, direct supervision and support, an application to the courts for a protection order, and/or removal of the child and placement in out-of-home care [13].

Out-of-home care placement (OOHC) can range from overnight care to long term placement (for example till 18 years of age). Placement can be with relatives other than parents (kinship care, where the government makes a payment to meet the costs of looking after the child), foster care (non-relative family placement again attracting government payment), residential care/group home. In Australia, the child remains with their birth family as a priority and, if removal is deemed necessary, returning the child to their birth family is a common goal [4]. Adoption is extremely rare and parental rights (of birth parents) cannot be compulsorily removed [1,13].

### Statistical analysis

The statistical analysis was conducted on an intention-to-treat basis. Means and frequencies were used to describe the socio-economic characteristics of the FPP and control women. Two sample t-test and chi square tests were used to compare characteristics between groups. Generalised linear modelling was used to estimate the relative risk (RR) of involvement with child protection by type of involvement (report, investigation, substantiation, OOHC placement) among FPP and control children.

Multivariate models included mothers’ socio-demographic characteristics, which were statistically significantly associated with the rate of involvement with child protection in the univariate analyses. We examined the possible differential effect of the FPP on child protection outcomes of maternal attributes identified elsewhere as important (mothers’ age group at time of the child’s birth and parity). We did this by grouping participants by age group and then by parity and conducting multivariate analyses (including socio-economic variables and parity or age group as appropriate). All analyses were performed using STATA 12.0 (Stata Corporation, USA).

## Results

### Mothers’ socio-demographic profile

Table 1 describes mothers’ socio-demographic characteristics. Characteristics found to be statistically significantly different between FPP and control mothers were age (FPP mothers being a mean 2.5 years younger), age distribution (greater proportion of mothers ≤20 years and fewer >30 years in the FPP group), parity (greater proportion of FPP mothers where the subject child was their first, 42.6% vs. 26.7%), and house move category (a greater proportion of FPP mothers with ≥1 house move/year, 20.6% vs. 15.8%). There was no difference in the sex or age distribution of children born to FPP and control mothers (data not included in Table 1).

**Table 1:** Demographic profile the FPP and control women.

Maternal attributes	Control		FPP		p-value
	(n = 563)		(n = 291)		
	No.	%	No.	%	
**Age (years)**					
Mean	25.6		23.1		<0.001
≤20	151	26.8	115	39.5	<0.001
21–30	281	49.9	146	50.2	
≥31	131	23.3	30	10.3	
**Parity**					
First child	149	26.7	124	42.6	<0.001
Second child	103	18.5	64	22.0	
Third child or more	306	54.8	103	35.4	
IRSAD* quintile at child birth					
Q1	173	35.2	89	32.5	0.069
Q2-4	301	61.2	182	66.4	
Q5	18	3.7	3	1.1	
**Employment status at child birth**					
Employed	70	19.7	43	20.0	0.935
Not employed	285	80.3	172	80.0	
**Rate of house moves (moves/year)**					
<0.5 move/year	353	62.7	152	52.2	0.013
0.5 –less than 1 move/year	121	21.5	79	27.1	
≥1 move/year	89	15.8	60	20.6	

* IRSAD Index of Relative Socio-economic Advantage and Disadvantage (IRSAD) from the 2011 Australian Bureau of Statistics–Socio Economic Indexes for Areas (ABS–SEIFA)

### Involvement with the child protection system

Involvement with the child protection system (CPS) from the child’s birth to 31/12/2016 among FPP and control children is reported in Table 2. This covers reports, investigations and substantiations expressed as mean annualised rate (to adjust for exposure period), and the mean annualised days in OOHC placement per child. These are unadjusted rates and as such comparison between FPP and control children are not the focus. What is of particular interest is which characteristics affect CP system involvement, best assessed from control group rates. Higher annualised rates were observed in children born to mothers of lower socio-economic quintile, to mothers not employed at the time of child birth and those experiencing a higher rate of house moves. All denote a more vulnerable population. No clear relationship was observed between CP system involvement and either age of mother or parity.

**Table 2:** Mean annualised rates (occasions per child-year) of involvement with child protection and annualised days in OOHC.

	Annualised rates						Annualised days	
	Report		Investigation		Substantiation		OOHC placement	
	**Control**	**FPP**	**Control**	**FPP**	**Control**	**FPP**	**Control**	**FPP**
All children	0.43	0.44	0.23	0.24	0.08	0.09	**15.4**	**8.4**^#^
**Age**								
≤20	0.48	0.35	**0.26**	**0.17**^#^	**0.08**	**0.04**^#^	10.1	6.3
21–30	0.43	0.48	0.22	0.26	0.09	0.11	17.9	11.4
≥31	0.40	0.52	0.25	0.29	0.10	0.10	16.4	1.6
**Parity**								
First child	0.40	0.30	0.21	0.15	0.06	0.04	**14.9**	**3.0**^^^
Second child	0.37	0.35	0.18	0.20	0.07	0.07	8.8	9.1
Third+ child	**0.48**	**0.64**^#^	0.27	0.35	0.11	0.13	18.2	14.4
IRSAD quintile*								
Q1	0.56	0.44	0.34	0.25	0.12	0.10	**27.2**	**9.4**^#^
Q2-4	0.43	0.39	0.21	0.20	0.08	0.07	11.0	7.8
Q5	**0.11**	**0.76**^#^	0.05	0.17	0.02	0.08	-	-
Employment status*								
Employed	0.17	0.28	0.08	0.10	0.02	0.03	3.1	0.02
Not employed	0.66	0.57	0.36	0.31	0.14	0.11	**23.2**	**10.8**^#^
**Rate of house moves**								
<0.5	0.36	0.35	0.19	0.17	0.07	0.07	**15.0**	**3.3**^^^
0.5–1.0	0.50	0.42	0.26	0.24	0.09	0.08	12.4	7.6
≥1.0	0.64	0.66	0.39	0.37	0.16	0.12	21.5	22.1

# denominator, includes only children with the specific type of child protection involvement

*At time of the child’s birth

^Statistically significantly different between Control and FPP groups (p-value < 0.05)

^#^ p-value > 0.05 & < 0.10

In terms of OOHC placement, the pattern in relation to IRSAD quintile was extremely pronounced as was employment, and house moves. Mean annualised days in care were lower in FPP than in control children. While we note these rates are not adjusted for baseline differences, this pattern was observed across almost all sub-categories. Differences were statistically significant for all children, children of first-time mothers, those born to mothers in the lowest socio-economic quintile, not being employed and those having the rate of house move of less than 0.5 per year.

**Fig 1:**
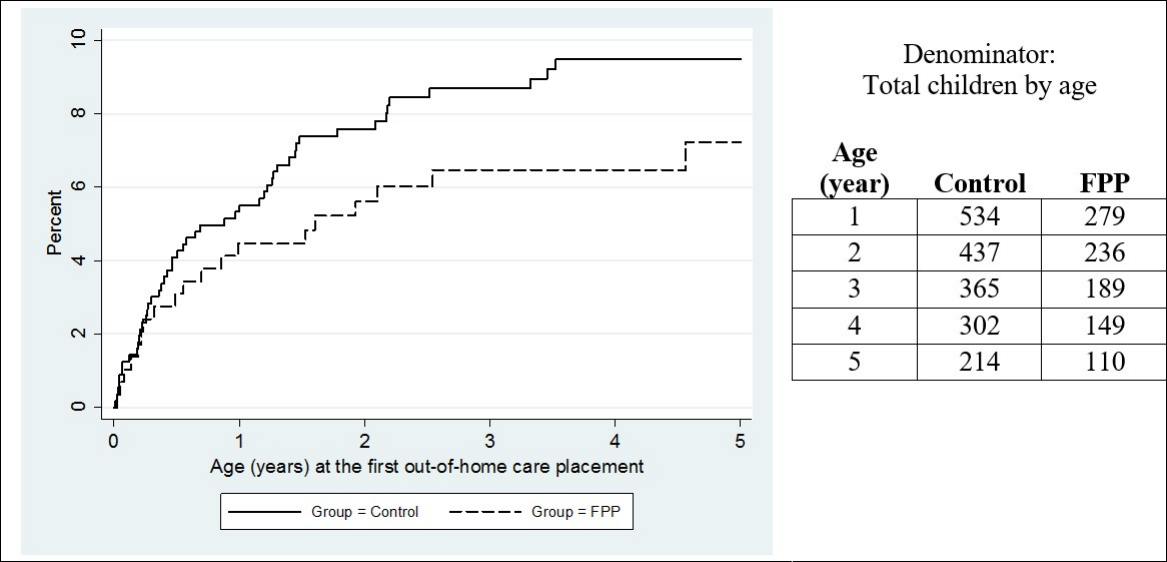
Unadjusted cumulative incidence out-of-home care placement—FPP and control children.

Unadjusted cumulative incidence of OOHC placement from birth is illustrated in Fig 1. Cumulative incidence was nearly identical in the FPP and control group to the fourth month of age, and then started to diverge, with greater incidence in the control than the FPP group. By five years of age, the incidence of out-of-home care placement was 9% among control children compared with over 7% among FPP children. The difference in the incidence curve of the FPP and control children, unadjusted for baseline differences, was not statistically significant.

In Table 3 we report the adjusted rate ratios of CPS events (reports, investigations and substantiations) and of annualised days in OOHC in FPP compared to control children. This is reported by mothers’ age group and parity and adjusted for IRSAD quintile, mother’s employment and rate of house moves. For children of mothers ≤20 years, the rates of a CP report, investigation or substantiation in FPP children were all substantially and statistically significantly lower than in control children (ARR_report_ = 0.49, ARR_investigation_ = 0.34, ARR_substantiation_ = 0.45). For children of older mothers, the rates of a CP event were not statistically significant different between control and FPP children. Among children of first-time mothers, the rates of a CP report, investigations or substantiation in FPP children were also substantially and statistically significantly lower than in control children (ARR_report_ = 0.50, ARR_investigation_ = 0.36, ARR_substantiation_ = 0.38). Where the child was not the first child of the mother, there was no significant difference in the rate of a CP event between the FPP and the control group.

**Table 3:** Adjusted effects of the FPP on the rate of child protection reports, investigation and substantiation by mothers’ age group and parity.

	Effects on annualised rates			Effects on annualised days
	Report	Investigation	Substantiation	OOHC placement
Mother’s age group*				
**Age: ≤20**				
Control	1.00	1.00	1.00	1.00
FPP	0.49 (0.29 to 0.82)	0.34 (0.19 to 0.64)	0.45 (0.21 to 0.96)	0.10 (0.02 to 0.48)
**Age: 21–30**				
Control	1.00	1.00	1.00	1.00
FPP	1.39 (0.41 to 4.69)	1.69 (0.39 to 7.25)	1.84 (0.32 to 10.62)	0.59 (0.02 to 20.42)
**Age: 31+**				
Control	1.00	1.00	1.00	1.00
FPP	1.05 (0.24 to 4.53)	0.90 (0.16 to 5.10)	0.89 (0.11 to 7.43)	0.02 (0.01 to 1.2)
**Parity^**				
**First child**				1.00
Control	1.00	1.00	1.00	0.06 (0.01 to 0.27)
FPP	0.50 (0.30 to 0.83)	0.36 (0.19 to 0.67)	0.38 (0.18 to 0.80)	
**Second child**				1.00
Control	1.00	1.00	1.00	1.17 (0.02 to 67.36)
FPP	1.59 (0.4 to 6.22)	2.39 (0.45 to 12.68)	3.90 (0.5 to 30.65)	
**Third child or more**				
Control	1.00	1.00	1.00	1.00
FPP	1.21 (0.37 to 3.99)	1.22 (0.28 to 5.28)	1.36 (0.22 to 8.33)	0.37 (0.01 to 11.43)

*Adjusted for mother’s parity, IRSAD quintile, employment status and rate of house moves.

^Adjusted for mother’s age group, IRSAD quintile, employment status and rate of house moves.

Regarding the effect of the FPP on annualised days in OOHC, the adjusted rate of days in OOHC was lower for FPP than control children across all age groups. This was statistically significant among children of mothers ≤20 years (Adjusted ratio = 0.10, 95%CI: 0.02 to 0.48). In terms of mother’s parity, only children of first-time mothers in the FPP group had statistically significantly fewer annual days in care than control children (Adjusted rate ratio = 0.06 (0.01 to 0.27).

Adjusted risk ratios of being ever involved with the CPS (ever reported, ever investigated, ever substantiated or ever placed in OOHC) between FPP and control children by mothers’ age group and parity are presented in Table 4. For mothers aged ≤20 years, and first-time mothers, risk ratios were less than 1 for all types of child protection system involvement but did not reach statistical significance.

**Table 4:** Adjusted risk ratio of ever involved with child protection system.

	Adjusted risk ratio of ever involved with child protection system			
	Report	Investigation	Substantiation	OOHC placement
Mother’s age group*				
**Age: ≤20**				
Control	1.00	1.00	1.00	1.00
FPP	0.77 (0.57 to 1.05)	0.72 (0.51 to 1.03)	0.75 (0.43 to 1.29)	0.53 (0.19 to 1.49)
**Age: 21–30**				
Control	1.00	1.00	1.00	1.00
FPP	1.02 (0.50 to 2.09)	1.17 (0.52 to 2.64)	1.31 (0.38 to 4.44)	1.03 (0.10 to 10.42)
**Age: 31+**				
Control	1.00	1.00	1.00	1.00
FPP	1.07 (0.47 to 2.45)	0.99 (0.38 to 2.55)	1.03 (0.25 to 4.23)	0.24 (0.01 to 5.99)
**Parity^**				
**First child**				
Control	1.00	1.00	1.00	1.00
FPP	0.79 (0.57 to 1.11)	0.84 (0.57 to 1.23)	0.82 (0.47 to 1.41)	0.44 (0.14 to 1.38)
**Second child**				
Control	1.00	1.00	1.00	1.00
FPP	0.89 (0.38 to 2.09)	0.97 (0.36 to 2.61)	1.29 (0.32 to 5.25)	1.83 (0.09 to 36.89)
**Third child or more**				
Control	1.00	1.00	1.00	1.00
FPP	1.06 (0.50 to 2.24)	1.04 (0.44 to 2.44)	1.14 (0.34 to 3.81)	0.65 (0.05 to 8.10)

*Adjusted for mother’s parity, IRSAD quintile and rate of house moves.

^Adjusted for mother’s age group, IRSAD quintile and rate of house moves.

## Discussion

As far as we are aware this is the first reported evaluation of child protection outcomes in an application of an adapted NFP model in a First Nation population in a remote setting. For children of young (aged 20 years or less) or first-time mothers, FPP children had statistically significantly lower rates of involvement with CPS (reports, investigations and substantiations), and fewer days in care per year than those not in the program.

Our findings are consistent with the results of the randomised trial of the NFP in New York State in the USA. Olds and colleagues reported that during the first 2 years of the children’s life, the percentage of children with reports of child abuse or neglect was 5% among those born to NFP teenage mothers (<19 years) compared to 15% among those born to control teenage mothers [14]. Among children of older mothers, these proportions among NFP and control children were very similar (6% and 5%, respectively) [14]. A randomised trial of the NFP in the Netherlands, reported that by age three and a half years, there was a higher proportion of children with reports of abuse and neglect among control (19%) than among NFP children (11%) [15].

An impact of child maltreatment is consistent with the underpinning theory. The FPP was adapted from the NFP model, which is grounded in theories including human attachment, human ecology and self-efficacy [16]. These promote the importance of a nurturing family environment, of women’s confidence in the face of obstacles and ability for sensitive, responsive, and engaged care-giving in the early years of the child’s life. The program content seeks to reduce risks and promote factors for positive child development outcomes, including a reduction in child abuse and neglect [16]. The model also has epidemiologic foundations for targeting disadvantaged women, but particularly low income, first time, teenage mothers.

In a systematic review of home visiting programs to prevent child maltreatment, Segal and colleagues found that programs with consistency in terms of the theory of change, the target population and the program content were more likely to achieve positive outcomes [17]. A further exploration of the FPP, the nature of program adaptions and how well they reflect the complex needs of the local Aboriginal community warrants separate investigation.

A limitation of this study is the use of a non-randomised study design, resulting in significant differences in the characteristics of control and FPP women. This trial design was dictated by the need to evaluate an existing program, and an unwillingness by Aboriginal ethics committees to support a randomised control trial of a program viewed as likely beneficial (Segal L 2017, telephone conversation, 13^th^ June). We have proceeded with the second-best approach of including all children of women who had been eligible for the program, the FPP group enrolled in the program and an eligible but non-referred group (excluding those who were referred but declined), then applying multivariable analyses to adjust for potential confounders. We acknowledge that bias can still arise from unobserved but important differences. We note that some bias in referrals is almost certain, with more first time and younger mothers more likely to be referred into and to take up the program, which we have also addressed through grouping the analysis by age and parity.

The use of child protection data within the NT jurisdiction means that CP involvement outside of the NT jurisdiction will not be captured. The potential for bias from this source is small as this applies to both control and FPP children.

Finally, there is always the risk of surveillance bias, given greater system contact of FPP children. It is uncertain what impact this might have. While it might increase the likelihood of a notification through greater service contact, if the FPP is known to be working with a family it might reduce notifications by others, if they feel the family is already being supported. That is, we hypothesise possible counteracting impacts that would likely leave a very small net effect [18].

Despite the study limitations, the consistency of our results with other nurse home visiting studies lends weight to the reported findings.

## Conclusion

The results of improved child protection outcomes of the FPP in first-time and young mothers, and in out-of-home care placement for all FPP children are a hopeful indicator of better mother child relationships. This might be expected to have a powerful influence on the life trajectory for children, especially in these highly vulnerable populations. While the observed outcomes are positive, further evaluation using a randomised design would be desirable to test these findings.
